# Collaboration Relations in Climate Information Production and Dissemination to Subsistence Farmers in Namibia

**DOI:** 10.1007/s00267-020-01383-5

**Published:** 2020-10-28

**Authors:** Chidiebere Ofoegbu, Mark New

**Affiliations:** 1 African Climate and Development Initiative, University of Cape Town, Rondebosch, 7700 South Africa; 2 Swedish species information centre, Swedish University of Agricultural Sciences, UPPSALA, Sweden

**Keywords:** Network analysis, Climate information, Knowledge brokering, Climate change adaptation, Subsistence farmers, Namibia

## Abstract

Although climate information can aid farmers’ capacity to adapt to climate change, its accessibility and adoption by subsistence farmers hinge on the collaboration between farmers and climate information providers. This paper examines collaborations among actors in the process of climate information production and dissemination in the Namibian agricultural sector. The aim is to investigate the extent to which subsistence farmers are integrated into the collaboration process and the impact of the collaboration on the nature and accessibility of disseminated information. Key informant interviews and a questionnaire survey were used for data collection. Using network analysis, we estimated the networks’ density, clustering coefficient, and degree centrality. The study found that both the climate information production and dissemination networks have a high overall clustering coefficient (78% and 77%, respectively) suggesting a high rate of collaboration among the actors in the networks. However, the frequency of interactions between the actors in both the information production and dissemination networks and subsistence farmers remains very low. Nearly all surveyed farmers reported that they meet with information providers only once in a year. The effect of this poor interaction is reflected in the poor occurrence of feedback learning, which is needed to optimize channels of information dissemination to subsistence farmers and enhance the robustness of disseminated information. We recommend innovative communication means via mobile phone, promotion of peer-to-peer learning, flexible collaboration relations with more space for feedback from the users of climate information, and more attention to long-term forecasts and their implications for adaptive actions.

## Introduction

The growing discourse on climate change integration into development policy agendas in developing countries (Milne et al. 2016; Hajer 1995) has warranted a rethink of the process of climate information generation and adoption in adaptation actions (Tall et al. 2018; 2013). Despite the steady growth in the production of information on climate risk and risk response strategy (Dilling and Lemos 2011; IPCC 2007), their accessibility and adoption have remained relatively limited. This, in turn, has limited their uptake and usage in decision making to support adaptation actions (Singh et al. 2017). This challenge is more pronounced in the agricultural sector of most developing countries and is very prevalent at the local scale among rural subsistence farmers (Angula and Kaundjua 2016). To promote the usability of climate information, all stakeholders must be involved in its production and dissemination (Naaba et al. 2019). To this end, there is a growing research interest in climate services to investigate options for the production and timely dissemination of climate information to inform policy and actions on adaptation (Harvey and Fisher 2013; Ziervogel and Zermoglio 2009).

Reducing the vulnerability of subsistence farmers to the impact of climate change will benefit from actions targeted at closing the gap between forecast generation and translation into information relevant to agricultural practices (Kalafatis et al. 2015; Feldman and Ingram 2009). This will entail the bridging of any existing inconsistencies between what climate forecasts generators perceive as useful, and what climate information users perceive as useful (Dilling and Lemos 2011). Doing this will require a shared vision of what climate information is usable in a given context (Stott and Huq 2014). This is supported by Sarewitz and Pielke’s (2007) postulation that the usability of climate information can be effectively pursued through policies and actions that encourage collaborations among information producers and users.

Until recently, the communication of information to local farmers in most developing countries follows the traditional pattern whereby scientific information generated through nationally managed information generation activity (with little or no input from local farmers) is disseminated via a top-down approach in which organizational structures and field extension workers are used to broker information to local users (Dayamba et al. 2018; Machingura et al. 2018; Feldman and Ingram 2009). The efficiency of this model of climate information dissemination is hampered by the disproportionate ratio of farmers to extension workers in most developing countries (Dayamba et al. 2018). Similarly, the limited or non-engagement with subsistence farmers in the process of climate information generation often results in the dissemination of climate information that is not relevant to the context of farmers, further hampering the efficiency of this model of climate information dissemination (Oreszczyn et al. 2010).

Hence, the growing research interest in innovative approaches to the process of climate information generation, and dissemination. The intent is to improve connections between holders of climate information across different scales and farmers, particularly at the local level (Cornell et al. 2013; Dilling and Lemos 2011). This concern focuses on organizational collaboration networks across scales among the actors engaged in the process of climate information generation and communication in the agricultural sector (Harvey and Fisher 2013). Whereas there is a growing number of studies on climate information generation and dissemination in developing countries, there is little or no information about Namibia, despite the importance of its agricultural sector in the national economy and the challenges the country is facing regarding climate change (Angula and Kaundjua 2016; United Nations Development Programme UNDP 2013).

Agriculture is one of the key sectors in Namibia. It supports the livelihoods of about 80% of the national population. Rain-fed farming is a key source of livelihood in Namibia. Rain-fed agricultural practices are highly sensitive to climate change (Angula and Kaundjua 2016; Cooper et al. 2008). Given the importance of the agricultural sector for food security in Namibia (Cooper et al. 2008), climate information forecasting, translation, and dissemination are priorities in the national adaptation action plan for the agricultural sector (Tall et al. 2018). Farmers in Namibia operate under highly risky environments. Providing them with timely climate information can be a good risk management strategy (Vogel and O’Brien 2006; Luseno, Winnie et al. 2003).

The process of climate information communication to farmers in Namibia is faced with the challenge of shifting to a more participatory approach that encourages collective learning among multiple actors of diverse disciplinary backgrounds (Wesley and Faminow 2014). Improving the connection between information holders and users is a key requirement for mainstreaming climate-smart agriculture (Dayamba et al. 2018; Vaughan and Dessai 2014). With this in mind, this study examines the extent to which collaborations among actors in the process of climate information generation and dissemination in the Namibian agricultural sector integrates rural subsistence farmers. The assumption here is that such integration will facilitate the production of climate information that is relevant to the context of subsistence farmers, and enable optimization of channels of information dissemination. This will, in turn, promote the usability and adoption of disseminated climate information in local farming practices.

The rest of the paper is structured as follows. After discussing the methodology in the next section, we present the results. The discussion positions the findings in the broader literature and addresses the policy implications. The conclusion synthesizes the findings and presents suggestions for further research.

## Methods

### Description of the Study Area

**Fig. 1:**
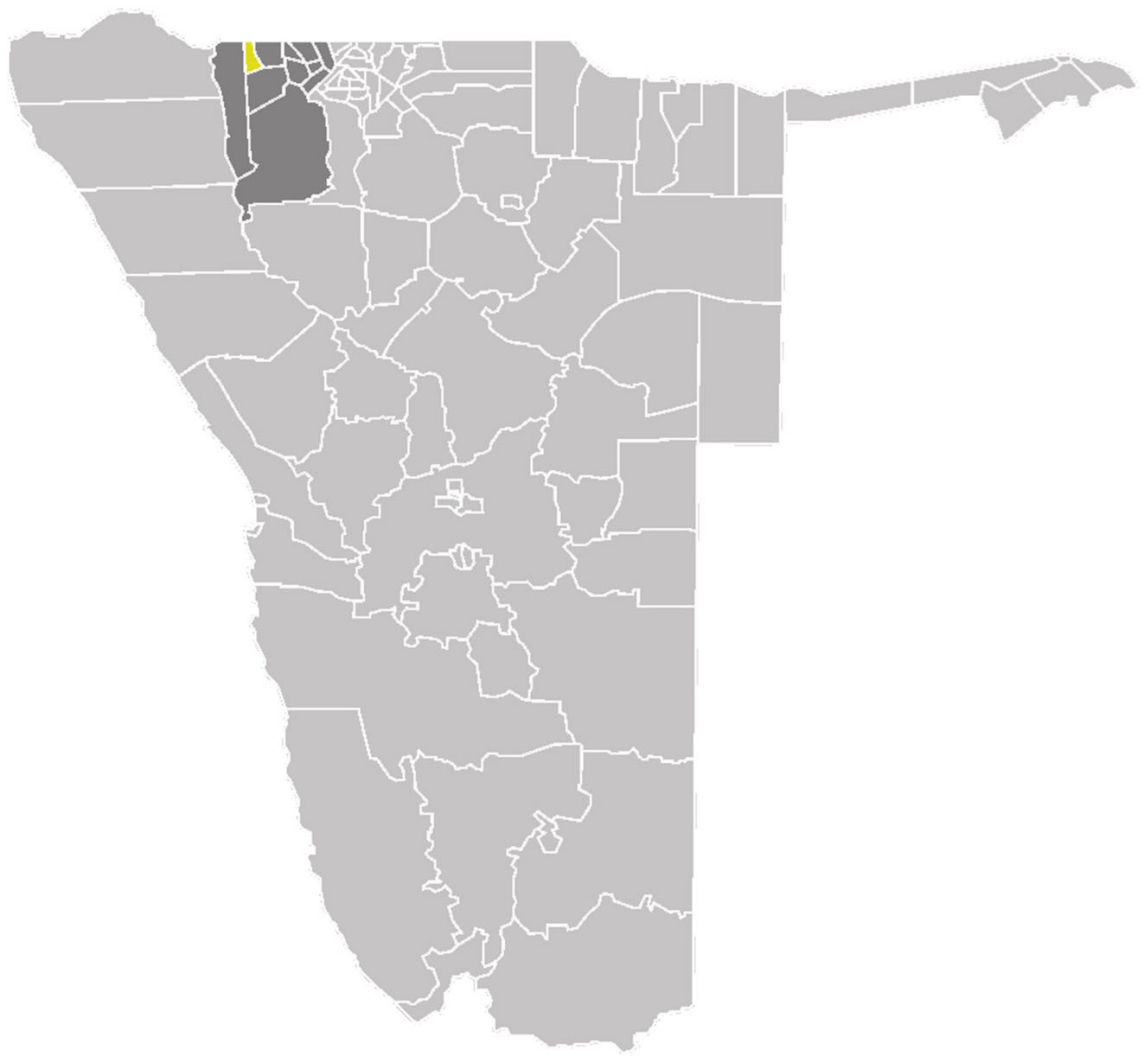
Onesi Constituency (yellow) in the Omusati Region (dark gray) (Source: Angula and Kaundjua 2016)

Namibia is located in Southern Africa. Its climate is characterized by sparse and erratic rainfall with over 90% of the total land area classified as either arid or semi-arid (Tadross and Johnston 2012). Most of the rain in the country is received in the northern part, which also has the highest population (Mendelsohn et al. 2002). Climate change is already affecting the fragile systems therein and projected impacts are grave unless urgent adaptive measures are taken (Reid et al. 2007). The UNDP (2015) ranked Namibia as the seventh most vulnerable country in terms of climate change-related agricultural losses. The study was conducted in the Onesi Constituency^1^ of Omusati Region (Fig. 1).

Subsistence farming is a major livelihood activity in the Omusati Region. Nearly all the farmers practice rain-fed farming (Angula and Kaundjua 2016). The subsistence farmers mostly grow grains (e.g., maize, sorghum, and millets), vegetables and also keep livestock, mostly cows, goats, and sheep. Omusati is largely made up of rural settlements that are facing environmental challenges like deforestation, land degradation, and scarce water resources (Angula and Kaundjua 2016). The dominance of subsistence farming and climatic challenges in the region makes it ideal for this study (Spear et al. 2015).

### Survey Approach and Data Collection

The survey targeted organizational collaborations in the generation and dissemination of climate information. The targeted participants were stakeholders in the intersecting field of agriculture and climate change. Climate information in the context of this study is defined as weather and climate forecasts and the associated climate risk warnings and risk response advisory services. Investigated forecasts are based on the following timescales: weather forecasts (days), seasonal forecasts (months), multi-year (1–5 years), intra-decadal (5–10 years), and decadal (10 years and above).

Data for the study is triangulated from three data-gathering procedures: literature review, key informant interviews, and a household questionnaire survey. Organizations that were mentioned in two or three of the data-gathering processes were used to define the key actors in the climate information network in Namibia (Table 1).

**Table 1:** Organizations included in the key informant interviews

No.	Acronym	Type	Name
1.	Airports	Government	Local and international airports in Namibia
2.	CBDRM	NGO	Community-Based Disaster Risk Management Initiative
3.	CDC	Government	Constituency Development Committee
4.	DRM	Government	Disaster Risk Management
5.	Farmers	CBO	Farmers Association
6.	Gobabeb	Research	Gobabeb Research and Training Centre
7.	IFRC	NGO	International Federation of Red Cross and Red Crescent Societies
8.	MAWF	Government	Ministry of Agriculture, Water & Forestry
9.	MET	Government	Ministry of Environment and Tourism
10.	MICT	Government	Ministry of Information Communication and Technology
11.	NAU	Government	Namibia Agricultural Union
12.	NMS	Government	National Meteorological Services
13.	RCS	NGO	Red Cross Society
14.	SARCOF	Research	Southern Africa Regional Climate Outlook Forum
15.	TAs	Government	Traditional Authorities
16.	Universities	Research	Universities

The key informant interviews focused on identifying the organizations’ collaboration in the generation and dissemination of climate information, and the timescale of climate forecasts that the organizations either generate, disseminate, or use in their operations. The key informant interviews also included questions on the organizations’ background and sphere of operation.

The household questionnaire was administered to farmers to contrast with the results of the key informant interviews. We surveyed farmers in the rural communities of Enongo and Omahenene of Onesi constituency to understand their perception of the relevance and usability of climate information communicated to them. The questionnaire included questions on channels of information communication, with whom they share the information, and perception of the information’s relevance and usability. We used a cluster random sampling procedure whereby the 16,613 inhabitants of Onesi constituency were clustered into ten communities (Namibia Statistics Agency (NSA) 2014). We then purposefully selected two communities (Enongo and Omahenene) based on the prevalence of subsistence farming systems and exposure to climate risk. We randomly interviewed 20 subsistence farmers in each of Enongo and Omahenene communities. Firstly, we obtained the names of all subsistence farmers in both Enogo and Omahenene communities with the assistance of the traditional leaders. We then randomly selected 20 farmers each in Enogo and Omahenene for the questionnaire survey. Prior to undertaking the survey, the questionnaire was pre-tested on ten subsistence farmers in Omusati town near the study communities. The reason was to check for errors and ambiguity, in order to improve the validity of the survey (Babbie and Mouton 2014). The demographic data of the farmers who participated in the questionnaire survey is presented in Table 2.

**Table 2:** Demographic profile of respondents

Demographic characteristics	Enogo (%)	Omahenene (%)
Age (years)		
≤35	17.8	15.5
36–47	18.5	12.7
48–58	21	18.2
59–69	21.7	27.3
≥70	21	26.4
Gender (%)		
Male	16.7	28.4
Female	83.3	71.6
Length of residency		
1–5	23.1	16.4
6–10	18.6	18.2
11–15	22.4	29.1
16–20	19.9	24.5
More than 20	16	11.8
Highest level of education (%)		
No formal education	64.6	73.4
Grade 11 or Lower	16.5	17.4
Grade 12(Matric, Std 10)	12.0	2.8
Post-matric diploma	3.8	4.6
Baccalaureate degree (s)	1.9	0
Postgraduate degree(s)	1.3	1.8

### Analysis

Data from the key informant interviews and the household survey was analyzed using both quantitative and qualitative techniques. Also, network analysis was used to analyze data on the collaborations between organizations in the generation and dissemination of climate information.

Data on respondents’ perceptions about their organizations roles in climate information generation and dissemination, influence in the collaboration network, the timescale of climate forecasts at organization’s disposal, perceived effect of organizations’ characteristics as either enabler or barrier of knowledge flow were extracted from the household survey and key informant interviews and analyzed using qualitative data analysis software (NVIVO 11) by coding and organizing the codes into themes (Strauss and Corbin 1990). Coding was applied to all transcripts at three levels (Strauss and Corbin 1990): initial/open coding, focused coding, and thematic coding. The transcribed interviews were coded line by line during the initial and open coding until no further new codes emerged (thematic saturation) (Ofoegbu et al. 2018). Coding focused on processes of information production, the content of disseminated information, and the nature of collaborations among the organizations.

The network analysis was aimed at exploring how the structure and cohesion of the collaboration networks act as either enabler or barrier of information flows. Respondents’ responses were coded as either 1 (presence) or 0 (absence) of a collaboration tie in either the generation or dissemination of climate information. Network data was analyzed and visualized using UCINET 6.0 and NETDRAW 2.0 software (Borgatti et al. 2002). The network structure was analyzed using the clustering coefficient. The network cohesion was analyzed using the density and degree centrality (see Table 3). Degree centrality is useful for identifying an organization’s influence in the network (Vance‐Borland and Holley 2011).

**Table 3:** Network properties analyzed

Network properties analyzed	Indices	Interpretations
Network structure	Clustering coefficient	Measures how close together a network is. The clustering coefficient of an actor is the density of its open neighborhood (Borgatti and Everett 2002).
	Core-periphery	Measures which actors belong in the core and which belong in the periphery of a network.
Network cohesion	Density	Measures the number of links, as a proportion of all possible links present in a network.
	In/out-degree centralization (used when the direction of ties is taken into account)	Measures the extent to which an actor is holding all the links in the network. An actor with high in-degree centralization can be characterized as *prominent*, while an actor with high out-degree centralization can be characterized as *influential*.
	Degree centrality (unidirectional)	Measures the number of links a node has as an indicator of dominance or power over information flow (Vance‐Borland, Holley 2011). Degree centrality is a useful metric to identify organizations that serve as a central source of information for the rest of the network.

## Results

### Types of Forecasts, Risk Warning and Advisory Services Being Communicated

The forecasts and the associated risk warning and risk response advisory services generated and communicated to actors in the agricultural sector in Namibia are based on weather and seasonal timescales. The major generator of these forecasts is the Namibian Meteorological Service (NMS), although there are other private institutes like the Gobabeb Training and Research Centre that are into weather forecasts generation and translation. As stated by the representative from NMS,

“We currently only generate weather and seasonal forecasts. Although we do not generate multi-year (1–5), intra-decadal (5–10), and decadal forecasts, we nevertheless are not blinded to the needs for such. We try to improvise when dealing with clients that may need such information”—Interview with NMS staff, Windhoek, October 2018.

The NMS rarely disseminates the forecasts directly to local farmers in rural areas. They mostly disseminate the forecasts via emails to actors in the banking sector, insurance companies, government ministries, NGOs/CBOs, and UN/multilateral organizations such as the FAO (Food and Agriculture Organization of the United Nations). All weather and climate risk forecasts disseminated by the NMS are packaged in probabilities. However, the NMS has provided training to actors working with farmers directly at the local community level (e.g., the regional office of the Red Cross Society) on how to interpret and translate the climate forecasts into suitable information for supporting the adaptation of farming operations to climate risk impact. As stated by the representative from NMS,

“Currently all climate risk forecast is packaged in the form of probabilities. However, in rare cases when we disseminate directly to non-expert e.g., individual local farmers, we try to explain the information to them in a way they can comprehend”—Interview with NMS staff, Windhoek October 2018.

Also, the representative from the regional office of the Red Cross society corroborated this statement by stating,

“NMS has trained us on how to interpret the forecasts, so we can use the forecasts in assisting the communities in terms of early warnings and preparedness for risks e.g., drought, flood and rainfall pattern/distribution”—Interview with Red Cross Society staff, Omusati September 2018.

The risk warning and risk response advice communicated to stakeholders in the agricultural sectors is mostly focused on the management of the risks of flood, drought, and erratic rainfall. Erratic rainfall as used here refers to the challenges/inconsistencies of the beginning and end of the rainy seasons, and the distribution of rainfall during the rainy seasons. Farmers are accordingly warned and advised on how to adapt their crops farming and livestock herding operations to these risks. An example of how weather and seasonal forecasts are applied in operational, tactical, and strategic decisions in the management of the risks of drought, flood, and erratic rainfall is presented in Table 4.

**Table 4:** Decision-making options associated with flood, drought, and erratic rainfall risks

Types of decision	Climate risks		
	Flood	Drought	Erratic rainfall
Operational	Early warning and preparation for a possible evacuation		The timing of crop watering and the fertilizer application period
Tactical		Decision-making on the type and variety of crop to be cultivated	
Strategic		Decision-making on the downsizing of livestock to coincide with the rangeland carrying capacity	

As can be inferred from Table 4, warning and advisory services communicated to relevant actors when dealing with the risks of flood, drought, and erratic rainfall are mostly focused on operational and tactical decision-making. The common type of advisory services communicated to farmers is early warning and preparedness. The preparedness of farmers includes buying supplemental feeds for livestock in readiness for drought and dry periods, the building of flood control structures and readiness for possible evacuation, and choosing the type and variety of crops resistant to drought.

### Collaboration Relation in Climate Information Production

The survey identified 11 organizations as key actors in the generation of weather and climate forecast concerning information on climate risk warning and risk response strategy for the Namibian agricultural sector. These organizations operate at regional and national scales (Table 5). Hence, the generation of weather and climate forecasts in Namibia mostly occurs at the national scale.

**Table 5:** Organizations in the production network according to the sphere of operation

Sphere of operation/category	Frequency	Members
Regional (SADC)	3	IFRC*, SARCOF*, SASSCAL*
National	8	Airports*, DRM*, Gobabeb*, MAWF*, MET*, NMS*, Red Cross*, Universities*

^*^SASSCAL Southern African Science Service Centre for Climate Change and Adaptive Land Management. For other acronyms see Table 1

The organizations operating at the national scale except for the DRM, MAWF, and MET are mostly self-sufficient in the generation of weather forecasts. However, they rely heavily on NMS for the generation of seasonal forecasts. Interestingly also the NMS depends strongly on their collaboration with SARCOF for technical expertize and assistance in the generation of seasonal forecasts. The Red Cross collaborates with the IFRC and NMS for the generation of weather and seasonal forecasts. The DRM, MAWF, and MET depend mostly on the NMS for the generation of both weather and seasonal forecasts. The Red Cross, MAWF, and MET are the key actors acting as boundary organizations in the translation of weather and seasonal forecasts into risk warning and risk response strategy for the Namibian agricultural sector.

**Fig. 2:**
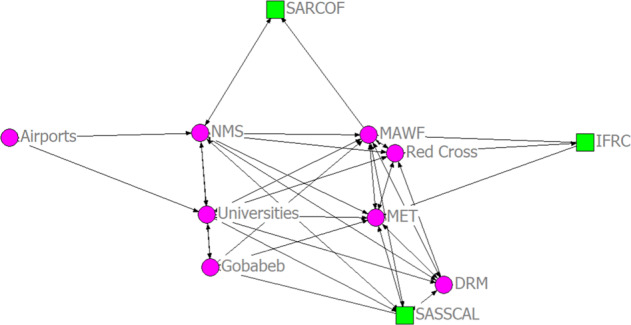
The climate information production network representing collaboration relations among stakeholders in the Namibia agricultural sector. Node shapes were based on an organization’s sphere of operation (circle: organizations operating at the national level; square: organizations operating at sub-national/regional level)

The climate information production network map (Fig. 2) gives a graphical illustration of the dynamics of the nature of relationships among the various organizations that constitute the climate information production network. The analysis of the collaborations in the process of climate information production reveals a complex interaction that shapes information flows and exchanges among the actors.

The climate information production network has an overall density of 0.509. This means that 51% of all possible ties in the network have been achieved. The overall clustering coefficient of the network was 0.783 (78%) and indicates how quickly information can spread within the network. Similarly, the centrality measure (degree centralization) indicates the role/influence of each organization in climate information generation. The individual clustering coefficient of the actors in the network along with their degree centrality is presented in Table 6.

**Table 6:** Clustering coefficient and degree centrality of the production network

		Clustering coefficient		Degree centrality	
SN	Organizations	Clustering coefficient (%)	Total number of possible ties	Degree	nDegree
1	Airports	1.000	1000	2000	0.200
2	DRM	0.867	15,000	6000	0.600
3	Gobabeb	0.900	10,000	5000	0.500
4	IFRC	1.000	3000	3000	0.300
5	MAWF	0.514	36,000	9000	0.900
6	MET	0.625	28,000	8000	0.800
7	NMS	0.472	36,000	9000	0.900
8	Red Cross	0.733	15,000	6000	0.600
9	SARCOF	1.000	1000	2000	0.200
10	SASSCAL	0.900	15,000	6000	0.600
11	Universities	0.607	28,000	8000	0.800

For acronyms see Table 1

Table 6 shows that the NMS and the MAWF have the highest degree centrality and can be regarded as the most influential actors in terms of climate information production. However, its clustering coefficient is relatively low, indicating a poor rate of collaboration with other actors in the network. In contrast, the MET and Universities who are also influential actors based on degree centrality have a moderately high clustering coefficient, indicating a higher level of collaborations in their operations. Interestingly, these organizations (MAWF, MET, and Universities) are mostly engaged in the translation of weather and climate forecasts into risk warning and risk response services. This suggests a greater level of collaboration among actors in the translation of forecasts into risk warning and advisory services than in the production of weather and climate forecasts.

### Collaboration Relation in Climate Information Dissemination

The survey identified 18 organizations as key actors in the dissemination of information on climate risk warning and risk response strategy concerning the Namibian agricultural sector. Twelve of these organizations operate at the national level (Table 7). Hence, the dissemination of weather and climate forecasts in Namibia mostly occurs at the national level and follows a top-down approach.

**Table 7:** Organizations in the dissemination network according to the sphere of operation

Sphere of operation/category	Frequency	Members
Regional/International	1	FAO*
National	12	The banking sector*, DRM*, Insurance sector*, MAWF*, MET*, MHSS*, MICT*, MOD*, MOF*, NMS, Red Cross*, Universities
Sub-national	1	RC
Local	3	CDC, Farmers, TA

^*^MHSS Ministry of Health and Social Services; *MOD* Ministry of Defense; *MOF* Ministry of Finance; *RC* Regional Council. For other acronyms see Table 1

The governmental organizations operating at the national level rely on their organizational structures for the dissemination of climate information to local farmers. The MAWF, for example, takes climate information from the national office through the regional/sub-national office to the local communities, whereby the final dissemination is done through the extension officers. The private sectors (banking and insurance sector) and the NGOs, particularly the international NGOs, operate similarly. However, the universities disseminate climate information directly to local farmers. Similarly, the organizations operating at the sub-national and local scale disseminate climate information directly with local farmers. In most cases, the regional and local offices operate in partnership with the CDC, traditional authorities, and farmers’ associations in disseminating climate information to local farmers.

**Fig. 3:**
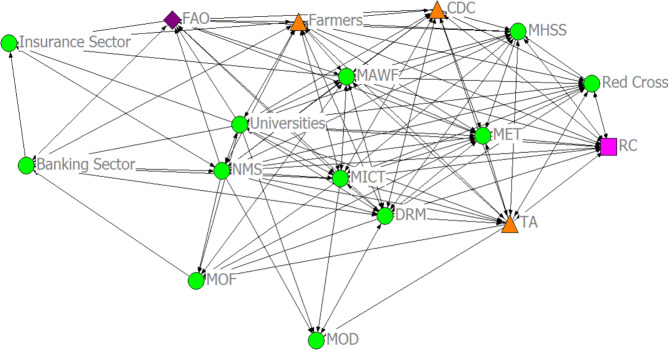
The climate information dissemination network representing collaboration relations among stakeholders in the Namibia agricultural sector. Node shapes were based on the administrative operation level of each stakeholder (circle: organizations operating at the national level; square: organizations operating at the sub-national level; triangle: organization operating at the local level; diamond: organizations operating at international level)

The analysis of collaborations in the dissemination network (Fig. 3) reveals complex interaction that shape climate information flows and communication to relevant stakeholders. The climate information dissemination network has an overall density of 0.629. This implies that 63% of all possible ties in the network are met. This indicates a moderately strong link (connection or relations) between the organizations that make up the network. The dissemination network has an overall clustering coefficient of 0.776 (78%). The clustering coefficient measures how close together actors in a network are. The closeness has implications on the ease of collaboration and flow of information among the actors. Hence, we can infer a good flow of information and collaboration among the actors in the dissemination network.

The individual clustering coefficient of the actors in the network along with their degree centrality is presented in Table 8.

**Table 8:** Clustering coefficient and degree centrality actors in the dissemination network

		Clustering coefficient		Degree centrality	
SN	Organizations	Clustering coefficient (%)	Total number of possible ties	Degree	nDegree
1	Banking sector	0.643	28,000	8000	0.500
2	CDC	0.894	66,000	12,000	0.750
3	DRM	0.652	105,000	15,000	0.938
4	FAO	0.755	55,000	11,000	0.688
5	Farmers	0.709	91,000	14,000	0.875
6	Insurance sector	0.833	15,000	6000	0.375
7	MAWF	0.698	91,000	14,000	0.875
8	MET	0.795	78,000	13,000	0.813
9	MHSS	0.902	66,000	12,000	0.750
10	MICT	0.657	105,000	15,000	0.938
11	MOD	0.950	10,000	5000	0.313
12	MOF	0.821	28,000	8000	0.500
13	NMS	0.600	120,000	16,000	1.000
14	RC	0.955	55,000	11,000	0.688
15	Red Cross	0.973	55,000	11,000	0.688
16	TA	0.756	78,000	13,000	0.813
17	Universities	0.600	120,000	16,000	1.000

For acronyms see Table 1

Based on degree centrality, the universities, NMS, and MICT appear to be the most influential actors in terms of climate information dissemination to local subsistence farmers in Namibia. Interestingly, these organizations (the universities, NMS, and MICT) have a moderately high clustering coefficient indicating a high rate of collaboration and quick diffusion of information through the network to the targeted end users. The organizations operating at the local level (traditional authorities, farmers/farmers associations, and the CDC) are also relatively influential based on their degree centrality.

### Organizational Collaboration Network and Connection to Rural Farmers

**Fig. 4:**
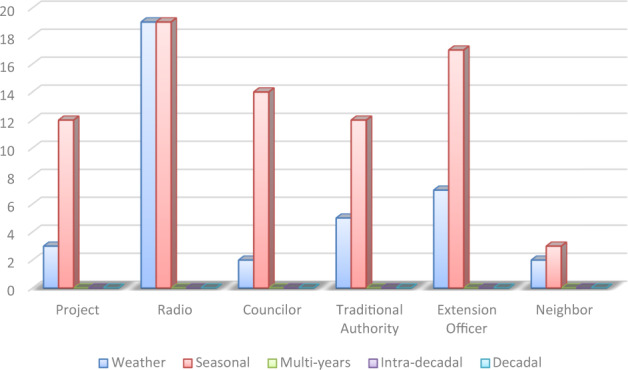
Actors and media mostly consulted for information on climate risk (*N* = 40)

The surveyed farmers’ in the Enongo and Omahenene communities of Onesi constituency in Namibia were asked to list five organizations that they consult most frequently when sourcing information on climate risk. We aggregated the result (Fig. 4) to present the frequency of mention of the actors and media most consulted by farmers.

Figure 4 indicates that weather and seasonal forecasts are the climate information of most interest to the local farmers. Climate projects also appear to be a significant source of climate information in the study area. Management and Project such as the ASSAR project, JICA rice project, and Land management project are being carried out in the rural areas in partnership with universities and sometimes with regional government or the constituency. These projects often provide training and disseminate practices on how to adapt farming operations to climate change impacts. Other important sources of climate information are radio stations, extension officers, traditional authorities, and the councilors.

**Fig. 5:**
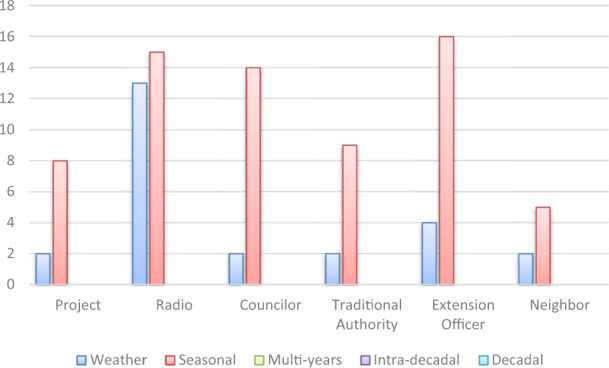
Actors and media mostly consulted for climate advisory information (*N* = 40)

The respondent were also asked to list the top five actors and media that provide them with advisory services on how to adapt their livelihood practices to climate change impacts. The intent was to discern whether climate information being provided to the people is done jointly to include both risk warning and adaptation advisory services, or whether they are provided separately. Results in Fig. 5 indicates that the majority of advisory services provided are based on the seasonal forecasts and the most common sources of these advisory service are radio and extension officers.

The local councilors and traditional authorities also play an important role in the delivery of advisory services to local farmers. Project-based advisory services are an important emerging source of climate information delivery. These projects often use new innovative media such as SMS (short message services) via mobile phone to deliver climate advisory services. All reported climate risk warnings provided to the people are focused on three dominant climate risks: early warning and preparation for flood risk, drought risk, and erratic rainfall impact. The advisory services vary but are focused on how to adapt livestock keeping and farming practices to the risks of flood, drought, and erratic rainfall. Table 9 summarizes the advice provided and shows that those that are most mentioned by the farmers include advice on cropping systems (when to weed); when to de-stock or give supplemental feed in the event of drought; on drought-resistant crops and varieties; and early warning in the case of flood risks.

**Table 9:** Farming advisory services provided and frequency of mention

Advice provided	Frequency
Assisting people to access relief materials	2
Advice on when to destock livestock in the event of drought	8
Advice on cropping system e.g., mixed cropping of mahangu with rice when to weed	11
Advice on crop choice/variety to plant when drought is predicted	6
Advice on when and type of livestock supplemental feeds to buy in times of drought	7
Advice on rain onset and when to start cropping cycle	2
Advice on choice of improved seeds to plant when good rains are expected	3
Advice on crop choice/variety to plant when projected rainfall is insufficient e.g., plant shorter maturing plant	3
Training on rainwater harvesting	2
Early warning-flood preparedness	6
Training of livestock keeping	3
Training on the preparation of cropping season for onset and offset of rain	1
Advice to choice and variety of crop to plant too much rain is expected	3

Respondents also indicated the type of climate advisory information they would like to receive, but which, they are not yet receiving. All the reported advisory information was essentially the type of information reported by other respondents as the type of information that they currently receive. The respondents were also asked to indicate the frequency of their meeting with climate information providers in their locality. 47% of respondents said that they meet once a year, 23% said every few months, 7% said every few weeks, 2% said once a week while 5% said every day.

## Discussion

The usability of climate information is a function of both how the information is produced and needed in different decision-making contexts (Dilling and Lemos 2011). We make comment on our findings on the production and dissemination of climate information in Namibia and how the climate information relates to farmers’ realities and needs.

### Climate Information Production and Dissemination and the Realities and Needs of Subsistence Farmers

In the case of Namibia’s agricultural sector, we observed that the main influential actor in forecast production (NMS) has the capacity for weather and seasonal forecasts production. Multi-year, intra-decadal, and decadal forecasts are currently not being produced. Consequently, all the climate risk warning and advisory services for the agricultural sector currently produced and disseminated in Namibia are based on weather and seasonal forecasts. These types of risk warning and advisory services are helpful for reactive adaptation action, but weak for anticipatory adaptation actions (Dayamba et al. 2018; Ofoegbu et al. 2018).

We observed that the type and nature of climate information demanded by subsistence farmers are mostly related to weather and seasonal forecasts. Two contemporary realities that drive this demand for weather and seasonal forecasts in the Namibian agricultural sector:

1. Most decision-making in the agricultural sector could benefit from integrated and targeted climate forecasts, such as the scheduling of planting or harvest operations, is made at a temporal scale that matches the timeline of shorter-term forecasts. Similar trends in the demand for short-timescale forecasts have been observed in the agricultural sectors in many African countries (Thornton et al. 2018; Mittal and Hariharan 2018; Tall et al. 2013; Ziervogel et al. 2009; Ziervogel and Zermoglio 2009).
2. The majority of subsistence farmers in the Namibian agricultural sector are poor and have limited capacity to invest in long-term adaptation action (anticipatory adaptation). Hence, the timescale of their management decisions are often based on a short timescale. Consequently, the farmer mostly demands forecasts of shorter timescale horizon (weather and seasonal).

These demand factors determine to a large extent the focus of climate information producers in Namibia on forecasts for short timescales (Naaba et al. 2019; Amissah-Arthur 2003). The dominant focus on shorter timescale forecasts poses a challenge for long-term anticipatory adaptation actions and the sustainability of the Namibian agricultural sector. From the national to the local level, there is a need for enhanced capacity to anticipate and predict the future scenario of the agricultural sector in the face of climate change, so that appropriate anticipatory adaptation action can be devised and implemented.

The lack of dissemination of information on risk warning and advisory services based on multi-year, intra-decadal, and decadal forecasts have limited the farmers to the cycle of reactive adaptation. Hence some of the farmers have adopted a highly flexible livelihood strategy and usually opt for either migration and/or off-farm livelihood practices in response to temporal climate change impacts (Cooper et al. 2008; Newsham and Thomas 2011). This type of adaptation action can negatively impact the development and sustainability of the agricultural sector. The onus is therefore on the NMS to develop the capacity for the generation of multi-year, intra-decadal, and decadal forecasts to facilitate the adoption of long term and anticipatory adaptation action in the agricultural sector.

### Organizational Collaboration Networks and Relevance of Climate Information to Local Farmers’ Context

Climate information usability can most effectively be pursued through deliberate policy design and implementation that unites information producers and users for co-production and sharing of climate information (Sarewitz and Pielke 2007). This is because climate information generated in isolation or independently is often insufficiently robust to meet the needs of the various user categories (Cramer et al. 2017; Cornell et al. 2013). In the case of the Namibian agricultural sector, we observed a mix of governmental, civic, and research-based organizations collaborating to generate and disseminate climate information to subsistence farmers. The collaborations among these organizations are shaped by many factors.

The information production network, has a slightly higher density than the information dissemination network signifying a higher rate of collaboration in information dissemination than in information generation. Unlike what was observed in the Kenya climate information network, where NGOs are playing an influential role in climate information generation and dissemination (Ofoegbu et al. 2018), both the production network and dissemination network in Namibia lack the strong influence of NGOs. Instead, we observed a moderately low influence of projects led by universities and research-based organizations in the generation and dissemination of climate information to subsistence farmers.

The MAWF and MET were the key boundary organizations playing a prominent role in the translation of weather and climate forecasts into risk warning information and advisory services relevant to the agricultural sector. Civic organizations like the Red Cross Society were also playing an important role as boundary organizations. These boundary organizations are critical to the usability of climate information in the Namibian agricultural sector in two ways. Firstly, they serve as brokers between the supply of and demand for climate information. They collaborate with the National Meteorological Service in the translation of climate forecasts to climate risk warning and risk response advisory services needed by actors in the agricultural sector. Secondly, they enhance communication among stakeholders. For example, MAWF, through its extension officers, facilitates the communication of climate information to subsistence farmers.

Although the study findings suggest a smooth flow of information in both the production and dissemination networks, the robustness of the communicated climate information to address the needs of subsistence farmers will hinge on how feedback and iterativity are maintained within the collaborations in both networks. A critical aspect of policy design to enhance co-production and dissemination of climate information is the creation of the conditions that facilitate iterativity (Dilling and Lemos 2011). Iterativity, as used here, refers to the number of meeting times among the actors in both the information production and dissemination network. It also includes the ease of access that subsistence farmers have to these actors. Maintaining iteration in the process of climate information production and dissemination in the Namibian agricultural sector will thus entail the promotion of continuous interactions and feedback loops for reflection and reinvention among the actors within both the production and dissemination networks.

The study findings showed that subsistence farmers mostly meet once a year with climate information providers in their communities. This low rate of meeting/collaboration with subsistence farmers is undoubtedly insufficient to maintain the robustness and acceptability of communicated climate information to subsistence farmers. Better communication between climate information producers and users requires engagement in a long-term dialog about each other’s’ needs and capabilities (Feldman and Ingram 2009). To achieve this, climate information producers must be committed to establishing opportunities for joint learning.

Though climate information generation and dissemination to rural farmers seem to cover all identified climate-related challenges in the communities, we observed some mismatch when farmers were asked to indicate types of information they wish to receive that they are not yet receiving. All the information mentioned by respondents was the same information identified by other respondents as the type of information that they are currently receiving. This is a strong indication that the frequency of meetings that currently exists between climate information providers and subsistence farmers is insufficient.

There is, therefore, a need to promote innovative means of enhancing the frequency and quality of interaction between subsistence farmers and information providers. This will help ensure that farmers have good knowledge and access to all available climate information in the country. To this end, promoting innovations like communication via mobile phone, promotion of peer-to-peer learning among subsistence farmers will be a good starting point (Yu et al. 2017). As argued by Feldman and Ingram (2009) knowledge networks should be intended for learning rather than knowing. Learning will require that collaboration relations in information production and dissemination have sufficient flexibility to encourage the diffusion of information across organizational type and individual spheres of decision-making.

## Conclusion

The translation of climate forecasts to tailored climate risk warning and risk response advisory services that is usable in the agricultural sector is a resource-intensive process that can be frustrated by the relatively small number of transdisciplinary scientists available. This study makes several contributions to both the science and practice of translating weather and climate forecasts into adaptation action targeting subsistence farmers’ adaptation to climate change. The understanding of the structure and cohesion of the collaboration network that exists between the actors operating in the generation and dissemination of climate information in the agricultural sector can operate as vehicles for enhancing the robustness of produced information and farmers’ access to the information.

The study has provided insights into (1) the structure and properties of climate information production and dissemination network in Namibia, and (2) the influence of the network structure and cohesion on information flows. Both the information production and dissemination networks displayed moderately high density and cohesion. Both networks indicate the existence of a smooth flow of collaborative and exchange relations among the different organizations that constitute the networks. Nevertheless, maintaining iterativity and feedback loop in the collaborations between organizations operating in climate information generation and dissemination is key to enhancing the usefulness of climate information to local farmers. Such reciprocity/feedback and link to the frequency of meeting with information producers are currently lacking. We, therefore, recommend further research on how the views and context of subsistence farmers can be better integrated into climate information production and dissemination. This should also entail research on the implications of social and gender differentiation on the process of climate information production and dissemination.

## Footnotes

1 The 14 regions of Namibia are divided into constituencies. There are currently 121 constituencies in Namibia. A constituency represent group of voters in a specified area who elect a representative to a legislative body.
